# Comparison of cystectomy and lobectomy of lung hydatid cyst in pediatrics: a retrospective study

**DOI:** 10.1097/MS9.0000000000001823

**Published:** 2024-02-28

**Authors:** Kaveh Behnia, Reza Shojaeian, Fasihe Mazandarani, Pegah Bahrami Taqanaki, Leila Ameri, Sepehr Shirzadeh, Mahdi Parvizi Mashhadi

**Affiliations:** Departments ofa General Surgery; b Pediatric Surgery; c Mashhad University of Medical Sciences; d Parsian Imaging Center, Mashhad; e Department of Pediatric, North Khorasan University of medical Sciences, Bojnurd, Iran

**Keywords:** Lung hydatid disease, paediatrics, cyctectomy, lobectomy

## Abstract

**Objective:**

Hydatid cyst is an endemic disease in Iran. The treatment of choice for paediatric lung hydatid cysts is surgical removal of the cyst. However, due to its high prevalence the risk of recurrence after the surgery, cystectomy with capitonnage, which preserves the lung tissue, is a favourable surgical approach compared to lobectomy. Herein, the authors compared the outcome of cystectomy and lobectomy of lung hydatid cysts.

**Methods and materials:**

This is a retrospective study conducted in the paediatric surgery department. Paediatric patients who had undergone surgery due to pulmonary hydatid cysts were enroled. The patients were divided into two groups including cystectomy and non-anatomic lobectomy. Then, the length of surgery, length of hospitalization, postoperative complications, and the time required to remove the chest tube were calculated in each group.

**Results:**

A total of 32 patients were enroled in this retrospective study. Age, sex, location, and size of cysts were not significantly different between the two groups. The duration of surgery in the lobectomy and cystectomy groups was 116.3±33.7 versus 116.1±28.2 min, respectively (*P*=0.53). Surgery complications including the need for blood transfusion, pneumothorax, need for bronchoscopy and atelectasis were not different between the study groups. The mean time for first chest tube removal was significantly different between the groups with the lobectomy group having a shorter time (*P*=0.02). The length of hospital and ICU stay were not different between the two surgical procedures. The time to remove the first chest tube was significantly higher in cystectomy compared to lobectomy (*P*=0.02).

**Conclusion:**

The complications and outcome of the cystectomy are comparable to the lobectomy technique. However, the cystectomy method has the advantage of preserving the lung tissue, therefore it’s a favourable technique in endemic areas for hydatid cysts where reoperation may be indicated.

## Introduction

Highlights

- Large hydatid cysts usually need lobectomy and cystectomy can be used as a noninvasive method in single and small hydatid cysts.
- Hydatid cyst has a high risk of recurrence after the surgery, therefore, cystectomy with capitonnage, which preserves the lung tissue, is a favourable surgical approach compared to lobectomy.
- The cystectomy method has the advantage of preserving the lung tissue, therefore it’s a favourable technique in endemic areas for hydatid cysts where reoperation may be indicated.

Hydatid cyst is a parasitic disease that is generally caused by Echinococcus granulosus. This disease is a serious health problem in endemic areas like Iran. Infection with Echinococcus granulosus is often observed in areas where human contact with dogs and sheep is high. The liver and lung are the most commonly involved organs; however, lung involvement is more common in paediatric patients^1–3^.

Unlike the liver, Hydatid cysts of the lung, are usually not calcified, and in cases where the cyst is intact and small, it is mostly asymptomatic. Lung hydatic cyst was shown to grow faster compared to the liver, due to the elastic nature of the lung tissue^4^. The clinical manifestations of these cysts depend on the location and size of the cyst. The most common symptoms of lung cysts are cough and chest pain. Rupture of the cyst into the adjacent bronchus is manifested by severe coughing and the discharge of sputum-containing mucoid fluid. Also, the leakage of the contents of the cyst to the outside may cause a hypersensitivity reaction and even lead to the death of the patient.

At present, surgery is the most common treatment option for lung hydatid cysts^5^. The most common surgical procedure for lung hydatid cysts is cystectomy with capitonnage. In this method, the walls of the evacuated pericyst cavity are approximated with multiple sutures. Cystectomy with capitonnage is highly recommended compared to lobectomy, especially in endemic areas, because there is a possibility of re-infection of children in endemic areas, so it is preferable to preserve the lung tissue. One of the important postoperative complications is the remaining or opening of the bronchopleural fistula from the cyst bed, which leads to air leakage or recurrence of pneumothorax after the patient is discharged^6^.

Various studies have investigated the complications of cystectomy with capitonnage surgical procedure^6,7^. However, so far, no study has been conducted comparing cystectomy and non-anatomic excision for pulmonary hydatid cysts in children in Iran. Therefore, the purpose of our study is to compare these two methods in order to find a selective method for the treatment of patients.

## Materials and methods

### Objective

The main outcome of this study was to compare the surgical complications between cystectomy with capitonnage and lobectomy methos in patients who underwent surgery for lung hydatid cyst. This project has been reported in line with the STROCSS criteria^8^.

### Study design and inclusion and exclusion criteria

This retrospective study was performed in our main paediatric teaching hospital. We included consecutive patients who were aged older than 18 years and had an intact, single, and peripheral hydatid cyst of the lung between 2022 and 2023. Patients who had pneumothorax due to perforated cyst were excluded from the study. Study data were obtained from the patient’s medical records. Patients’ information remained confidential. The study protocol was approved by the ethical committee.

### Surgical technique

Based on the surgical procedure participants were divided into two groups including cystectomy with capitonnage and lobectomy. In the first group, the cyst was drained, the germinal lamina was removed, the pericyst was left, and the bronchial fistulas were closed. In the second method, which is mostly performed in peripheral cysts, complete removal of the cyst and pericyst with a margin of the healthy surrounding tissue was performed, which is often done in the form of segmentectomy or lobectomy.

### Data extraction

Patients‘ demographic data and cyst characteristics were recorded. Participants were also followed up for 6 months to monitor the surgery complications including pneumothorax, the need for bronchoscopy, the need for blood transfusion during or after the surgery, lung atelectasis, and the need for chest tube replacement. To evaluate the outcome of the surgery, the length of hospital and ICU stay, and time to first and second chest tube removal were recorded.

### Postoperative follow-up

Postoperatively, all cases received prophylactic empirical antibiotic treatment with amoxicillin-clavulanic acid. Chest tubes were removed once there was no detectable air leak, and drainage was below 100 ml within a 24-h period. Following discharge, all patients underwent albendazole treatment at a dosage of 15 mg/kg/day for two cycles of 15 days each. A drug-free rest period of 10 days was implemented between the two cycles.

### Statistical analysis

Qualitative data are presented as frequency (%) and quantitative data are presented as mean ± standard deviation. To compare the categorical data χ^2^ test was used. To compare the quantitative data between study groups independent sample *t*-test was used. Data were analyzed using SPSS v 23. *P* value less than 0.05 is considered statistically significant.

## Results

A total of 32 patients were entered in this retrospective study of which 17 patients underwent lobectomy and 15 underwent Cystectomy. The mean age of cystectomy patients was 7.6±2.1 years and lobectomy patients were 6.6±2.5 years (*P*=0.24). Sex, location of cysts, and cyst size were not significantly different between study groups (Table 1).

**Table 1:** Comparison of demographic data, location, and size of cysts between the study groups

Type of surgery variables	Cystectomy (*N*=15)	Lobectomy (*N*=17)	*P*
Age (year)	7.6±2.1	6.6±2.5	0.24^a^
Sex (female), *N* (%)	6 (40)	8 (47.1)	0.69^b^
Location of cysts, *N* (%)			
Right lung	7 (46.7)	10 (58.8)	0.49^a^
Left lung	8 (53.8)	7 (41.2)	
Cysts size (mm)			
Length	38.5±12.6	42.7±11.5	0.26^b^
Width	28.6±7.3	34.4±7.4	0.06^b^

Data are presented as mean±standard deviation or frequency (%).

a χ^2^ test.

b Independent sample *t*-test.

We found no significant difference in the duration of surgery between the cystectomy and lobectomy groups (*P*=0.53). Surgery complications including the need for blood transfusion (*P*=0.44), pneumothorax (*P*=0.66), need for bronchoscopy (*P*=0.68), lung atelectasis (*P*=0.99), and need for chest tube replacement (*P*=0.66) were not significantly different between the two surgery approaches. The mean time for first chest tube removal was 5.5±2.0 days in the cystectomy group and 4.7±3.4 days in the lobectomy group, which were significantly different (*P*=0.02). However, we found no difference in the time for second chest tube removal between the two groups (*P*=0.70). The mean length of hospital and ICU stay were not different between the two groups (Table 2). No recurrence had occurred in any of the patients.

**Table 2:** Comparison of duration, complications, and outcome of the surgery between the two types of operations

	Cystectomy (*N*=15)	Lobectomy (*N*=17)	*P*
Duration of surgery (min)	116.3±33.7	116.1±28.2	0.53
Surgery complications, *N* (%)			
Blood transfusion	3 (20)	6 (35.3)	0.44
Pneumothorax	2 (13.3)	4 (23.5)	0.66
Need for bronchoscopy	4 (26.7)	3 (17.6)	0.68
lung atelectasis	5 (33.3)	4 (29.4)	0.99
Chest tube replacement	2 (13.3)	4 (23.5)	0.66
Surgery outcome			
Time to first chest tube removal (day)	5.5±2.0	4.7±3.4	0.02
Time to second chest tube removal (day)	8.6±3.1	10.6±5.2	0.70
Length of hospital stay (day)	11.8±4.9	12±5.9	0.97
Length of ICU stay (day)	1.9±0.7	2.23±1.9	0.65

## Discussion

Treatment of hydatid cyst has remained a challenge for the surgeons. Surgical removal is still the treatment of choice in both children and adults. A hydatid cyst surgery may cause complications in 5–13% of the cases. These include pneumothorax, pleural effusion, hydro-pneumothorax, empyema, tension pneumothorax, pleural thickening, bronchopleural fistula, rapped lung, and secondary pleural hydatid disease. The rate of complications is even higher in children and is reported to be 12.9–19%^9^. Surgical removal involves different techniques including cystotomy, cystotomy–capitonnage, wedge resection, cystectomy, and lobectomy^10^.

We compared two surgery methods regarding hydatid cyst removal. We found that the time to expel the chest tube was significantly higher in the cystectomy group. However, the mean number of inserted chest tubes and the lung cyst size were higher in the lobectomy group, but not statistically significant. The rate of complications like pneumothorax, reinsertion of chest tube, and blood transfusion were higher in the lobectomy group. Moreover, the cystectomy group had a higher rate of lung compression and an indication for bronchoscopy. Still, these were not statistically significant.

Khan *et al.* ^11^ compared the efficacy of different surgery methods in the case of hydatid cysts. They reported that cystotomy and capitonnage have the highest efficacy, which was 98%. However, postoperative prolonged air leak was the most important complication of this method. They also reported that the efficacy of lobectomy was higher than the of cystectomy. Also, the rate of complications like atelectasis and lung collapse was higher in the cystectomy group. In line with their findings, we found that the cystectomy group had a higher rate of lung compression. However, the study population of Khan *et al.* ^11^ comprised both children and adults. Bagheri *et al.* ^12^ also conducted another similar study that compared three surgery methods including cystectomy, lobectomy, and Ugon method. They reported that the Ugon method had the highest rate of complication, which was 46.15%, followed by lobectomy (15.38%), and cystectomy (10.25%). Our study also showed that cystectomy group had lesser complications compared to the lobectomy cases.

Tahir *et al.* ^13^ presented that children are more prone for developing widespread disease and thus the rate of lobectomy is high in these cases. They reported that 68.96% of their studied cases underwent cyst resection, 3.44% needed partial lobectomy or segmentectomy, and 27.58% experienced lobectomy. In our study, 53.1% underwent partial lobectomy and the remaining had cystectomy.

The use of surgery method in hydatid cyst removal should be weighed out by the rate of recurrence, re-infection and complications. Moreover, it should be considered that large cysts usually need lobectomy and cystectomy can be used as a noninvasive method. Cystectomy may be considered as a less invasive method and may be treatment of the choice in small cysts^14,15^.

Our study had some limitations. First, we assessed short-term complications of the surgery. Second, we compared only the two of the surgery methods regarding hydatid cyst removal. Lastly our study did not include complicated cysts. However, the study was among few investigations that was conducted on paediatric population.

## Conclusion

In summary, our study revealed no significant differences in the duration of surgery, mean lengths of hospital and ICU stay, and various surgery-related complications between the cystectomy and lobectomy groups. Notably, the need for blood transfusion, pneumothorax, bronchoscopy, lung atelectasis, and chest tube replacement showed no statistically significant distinctions between the two surgical approaches. While the mean time for first chest tube removal exhibited a significant difference, the time for the second chest tube removal did not. Hence, since the possibility of re-infection is high in endemic areas, given the lack of an statistical significant difference in adverse events or hospital stay, our findings highlight the potential advantages of employing cystectomy with capitonnage as a less invasive surgical technique, which preserves the lung tissue for possible future surgeries in children with lung hydatid cyst.

## Ethical approval

The study protocol was approved by the ethical committee of Mashhad University of Medical Sciences with the ethical code of: IR.MUMS.IRH.REC.1402.095.

## Consent

Written informed consent was obtained from the patient for publication of this case report and accompanying images. A copy of the written consent is available for review by the Editor-in-Chief of this journal on request” Written informed consent was obtained from the patients' parents/legal guardians for publication and any accompanying images. A copy of the written consent is available for review by the Editor-in-Chief of this journal on request.

## Sources of funding

This research did not receive any specific grant from funding agencies in the public, commercial, or not-for-profit sectors.

## Author contribution

Study concept or design: M.P.M., K.B. Data collection: K.B., R.S., F.M., P.B.T. Supervision: M.P.M. Data analysis or interpretation: K.B., R.S., F.M., P.B.T., L.A., S.S., M.P.M. Writing the paper: K.B., R.S. Revision: M.P.M., A.V.

## Conflicts of interest disclosure

None.

## Research registration unique identifying number (UIN)

researchregistry9557.

## Guarantor

Mahdi Parvizi Mashhadi.

## Data availability statement

All the required data are available in the manuscript.

## Provenance and peer review

Not commissioned, externally peer-reviewed.

## Previous communication

None.
